# “Who Are at Higher Sexual Risk?” Latent Class Analysis of Behavioral Intentions among Spanish Adolescents

**DOI:** 10.3390/ijerph18041855

**Published:** 2021-02-14

**Authors:** Alexandra Morales, Samuel Tomczyk, Mireia Orgilés, José Pedro Espada

**Affiliations:** 1 Department of Health Psychology, Miguel Hernández University, 03202 Elche, Spain; morgiles@umh.es (M.O.); jpespada@umh.es (J.P.E.); 2 Department Health and Prevention, Institute of Psychology, University of Greifswald, 17489 Greifswald, Germany; samuel.tomczyk@uni-greifswald.de

**Keywords:** HIV, latent class models, teenagers, sexual health, reproductive health

## Abstract

Consistent condom use tends to be limited in youth, which makes this group especially vulnerable for sexually transmitted infections (STIs) and unplanned pregnancies. It is known that sexual risk may vary as a function of behavioral intentions (e.g., condom use intention or having sex under the influence of alcohol), but no studies have yet characterized the sexual risk profiles considering behavioral intentions. This study utilizes latent class analysis (LCA) to explore the subtyping of behavioral intentions related to sexual risk in a community-based sample of adolescents aged 14 to 16 years from Spain. Multinomial logistic regression was used to assess the association between class membership and participants’ sociodemographic variables (sex, age, educational level, socioeconomic status, and family situation), and behavioral variables (sexual experience and percentage of condom use). Among the 1557 participants, four latent classes of risk were identified: “Condom + drugs”, “abstinent”, “condom + no drugs”, and “no condom + drugs”. Differences in adolescents’ sex, age, educational level, sexual experience, and condom use across latent classes were found. Findings highlight opportunities for psychologists, educators, and health-care providers to promote condom use in adolescents with differing sexual risk profiles. Increased understanding of behavioral intentions among adolescents may help to reduce sexual risk behaviors in this group.

## 1. Introduction

Sexually transmitted infections (STIs) are among the most common conditions and may compromise people’s health and lives worldwide [1]. Apart from the physical complications associated with STIs (e.g., genital ulceration, pelvic inflammatory disease, and infertility), individuals with STIs may experience shame, stigma, vulnerability, stereotyping, and gender-based violence [1,2]. There are 376 million new infections per year with one of four curable STIs: Chlamydia, gonorrhea, syphilis, and trichomoniasis, which means that more than 1 million people are acquiring an STI daily [1,3]. It is estimated that more than 500 million people have a genital infection with herpes simplex virus (HSV), and more than 290 million women have a human papillomavirus (HPV) infection [4,5]. People who have an STI are at higher risk of contracting HIV, so far, without a cure, although antiretroviral treatment has made it a chronic condition. Spain holds the global rate of new HIV diagnoses (8.82 per 100,000), higher than the global average in Central Europe (3.2 per 100,000) and West Europe (6.4 per 100,000) [6,7]. The trend for other curable infections, such as gonorrhea and syphilis, has been increasing since the 2000s [8]. Most of the cases diagnosed with STIs occur in young adults, and these infections tend to be asymptomatic. This may explain that about 46.7% of cases are diagnosed late (understood as the presence of a CD4 cell count less than 350 cells/µL) [8]. Based on this, adolescence, when the first intimate relationships often begin, is considered a critical stage to prevent STIs. Unplanned pregnancies are another risk that adolescents are exposed to. Becoming a parent in adolescence may compromise the mother’s psychosocial development (e.g., educational level, work opportunities, socioeconomic status, etc.) and health (e.g., maternal anemia, pre-eclampsia, and preterm birth) [3,9,10].

Condoms offer one of the most effective methods of protection against STIs and unplanned pregnancies when used correctly and consistently [3]. The condom is the most used protection method in sexual intercourse in youth [11]. A study of Health Behavior in School-aged Spanish Children (HBSC), which involved over 10,000 participants from Spain aged 11–18, evaluated in 2002, 2006, and 2010, concluded that condom use has decreased over time in Spanish adolescents aged 15–18, from 90.9% who used a condom in their last intercourse in 2002 to 74.4% in 2010 [12]. Although the percentage of condom use is relatively high, consistent and correct condom use is much lower (20–50%) [13,14]. Condom use intention has been considered the best predictor of condom use, according to theoretical models such as the theory of planned behavior (TPB) [15,16,17]. Attitudes, perceived norms, and perceived control are predictors of behavioral intentions. The relationship between condom use intention and the use of this protection method has been proven in meta-analytic and empirical studies with different populations [18,19,20]. In Spain, a study that tested three sociocognitive models to predict condom use in adolescents showed that the TPB was most appropriate and that condom use intention was a significant predictor of the frequency of condom use in adolescents (*β* = 0.61, *p* < 0.001) [21].

In the prediction of condom use, one of the most studied risk factors has been drug use, especially alcohol [22,23]. Sex under the influence of alcohol and other drugs is considered a risk behavior because it is associated with behavioral disinhibition, a reduction in risk perception, and a loss of self-control [23]. In a study in which slightly more than one thousand Spanish adolescents aged 14 and 18 years old participated, those who reported having had sex under the influence of alcohol were less likely to report consistent condom use, more likely to engage in vaginal sex, oral sex, and anal sex, and presented a more negative attitude toward condom use when there are obstacles to its use, compared to those who reported not having mixed sex and alcohol [24]. Consistently, a growing number of studies concluded that young people who have sex under the influence of drugs were more likely to contract an STI and/or conceive an unplanned pregnancy, both associated with lower condom use [22,25]. Although some studies have found no clear relationship between drug use and risky sexual behaviors [26], considerable evidence suggests that youth who have sex under the influence of alcohol and other drugs are more likely to engage in sexual risk behaviors, such as not using a condom [22,25,27,28].

Risk factors associated with condom use have been widely studied in samples of adolescents and young people (see [23]), but these factors are usually approached separately, and the different risk profiles that may occur are not considered. For example, adolescents may intend to seek a condom in case they have sex, intend to do so, and may also intend to have sex under the influence of alcohol and drugs. Another possible profile could be that of adolescents who do not intend to use condoms but also do not plan to have sex under the influence of alcohol or other drugs. Although several of these combinations are possible and may differentially affect the risk of contracting an STI and/or conceiving an unplanned pregnancy, to our knowledge, there are no studies that have explored such profiles of behavioral intentions related to sexual risk in adolescents. Past research has focused on analyzing separately sexual risk factors in adolescents (e.g., condom use intention) [21,23], but subtyping behavioral sexual risk intentions has not been explored using person-based methods, such as latent class analysis (LCA).

Methods such as the LCA examine the heterogeneity of risk factors associated with health behaviors (e.g., using a condom during sexual intercourse) and group individuals based on their individual differences. LCA is a statistical technique that aims to classify individuals into mutually exclusive groups, which share similar characteristics [29,30]. LCA identifies unobservable groups and allows a better understanding of the concurrent impact of exposure to several risk factors, as well as the antecedents and consequences of complex attitudes and behaviors so that interventions can be tailored to specific groups and a greater benefit can be obtained [29]. In this field, LCA can provide information on the level of sexual risk in adolescents and help to illustrate different effects and special groups for the prevention of and approach to risky sexual behaviors, considering adolescents’ intentions of engaging in healthy and unhealthy behaviors.

To address this gap, this study used LCA to explore the subtyping of behavioral intentions related to sexual risk in a sample of Spanish adolescents aged 14 to 16 years old. To determine the existence of significant groups of individuals with different levels of sexual risk, LCA was carried out using their self-reported behavioral intentions (intention to seek condoms, intention to negotiate condom use with the sexual partner, condom use intention, and intention to have sex under the influence of alcohol and/or other drugs) to identify sexual risk profiles in adolescents. Furthermore, we aimed to determine the relationship among behavioral intentions (to seek condoms, use a condom, negotiate condom use, to have sex under the influence of alcohol and/or drugs), the latent classes and demographic variables (i.e., adolescents’ sex, age, educational level, socioeconomic status, and family situation), and adolescents’ sexual behavior (being sexually experienced or not, and percentage of condom use). We hypothesize that analyses will reveal latent classes of adolescents with different levels of sexual risk according to their intention to engage in sexual risks such as condom use intention (if they have sex) and intention of having sex under the influence of alcohol or drugs. According to previous studies [31,32], it was hypothesized that males (compared to females), older participants (compared to the younger ones), and consequently those belonging to a higher level of education (compared to those belonging to a lower level of education) would be more likely to belong to profiles with higher sexual risk. No conclusive results were found regarding the relationship between sexual risk and socioeconomic status or family situation, so no hypotheses were formulated.

## 2. Materials and Methods 

### 2.1. Participants

The participants were 1557 community-based adolescents (51.2% were boys) from the 9th and 10th grades in Spain. With the permission of the principals and informed consent by parents, we obtained a sample from 18 different middle schools in four Autonomous Communities, located in the north, east, south-east, and south of Spain. Table 1 presents detailed participant characteristics.

### 2.2. Measures

#### 2.2.1. Demographic Variables

Demographic variables of interest included: Sex (male vs. female), age (in years), educational level (9th grade, 10th grade, curriculum adaptation—level 1, and curriculum adaptation—level 2), socioeconomic status (low, medium, and high), and family situation (married parents, divorced parents, unmarried parents living together, single parent, and orphan). Response options are presented in Table 1. Socioeconomic status was measured with the family affluence scale by Boyce et al. [33]. It evaluates a family’s economic well-being with four items: The number of cars and computers a family possesses, possession of a bedroom to oneself, and the number of family vacation periods taken during the preceding 12 months.

#### 2.2.2. Behavioral Intentions

A 5-item scale to evaluate intention to engage in safer sexual behavior in the next 12 months was used [34]. Each item is rated on a scale ranging from 1 (definitely not) to 5 (definitely). It comprises two subscales: (1) The intention to acquire, use, and negotiate condom use with a sexual partner (α = 0.80); and (2) the intention to have sex under the influence of alcohol and other drugs (α = 0.75). In the present study, the ordinal alpha was 0.82 for both subscales.

#### 2.2.3. Sexual Behavior

The following behaviors were assessed: (a) Type of sexual experience (petting, vaginal sex, oral sex, anal sex, and mutual masturbation) (yes/no), and (b) frequency of condom use (range of percentage: 0–100).

### 2.3. Procedure

This cross-sectional study is part of a broader project aiming to explore sexual behaviors in Spanish adolescents, which was approved by the ethics committee of the Miguel Hernández University (DPS-JPE-001–10). Eligible participants were students enrolled in the 9th and 10th grades (14–16 years old). Eighteen middle schools located in the north, south, east, and southeast of Spain participated, after the school principals had agreed. There were about 300 participants (about 100 per school) recruited in each of the five provinces involved in the study. More information about the recruitment process can be found at Espada et al. [34]. Parental written informed consent was obtained, after explaining the objective of the study and ensuring the confidentiality of the data. The assessment was undertaken in groups of approximately 30 students and it was online using Google Forms. The questionnaire took approximately 45 min to complete, and a trained research assistant was present to aid participants. No participant withdrew from the study after they initiated the survey. No incentives were provided to the participants who completed the survey.

### 2.4. Data Analyses

To examine intentional patterns among adolescents, latent class models were computed via Mplus 8 [35]. Originally assessed on a five-point scale, intention was dichotomized for this analysis due to its skewed distribution (see Table 1). Consequently, the indicators reflected negative (“0,” definitely not, probably not) and positive (“1,” probably, very probably, definitely) intentions toward sexual behaviors. This led to 14.3% to 95% of adolescents reporting positive intentions regarding acquiring (79.5%), using (95%), and negotiating (92.8%) condoms, as well as combining sexual activities with alcohol (38.2%) or other drugs (14.3%). Overall, missing data were low (0.7%; 11/1557) and random.

Latent class estimation was an iterative process, estimating models and comparing the model fit between one and six latent classes. A robust maximum likelihood estimation (command MLR in Mplus) was chosen with 200 initial random starts and a logit link function. This estimator is robust in estimating latent class models with randomly missing data [36]. The model fit was evaluated based on the interpretability and theoretical tenability of the classes, as well as statistical indicators of goodness of fit, sparseness, and classification quality [37,38,39]. Therefore, the bootstrapped likelihood ratio test (BLRT) captured the overall fit by comparing the estimated model with k classes to a model with k-1 classes using 50 random starts with 20 bootstrap draws for each comparison. A significant test indicates a preference for the model with k classes over the model with k-1 classes. Lower values for the Akaike Information Criterion (AIC) and the sample-size-adjusted Bayes Information Criterion (BIC) indicate model sparseness. Finally, average latent class probabilities (ALCP) and entropy reflect classification quality (i.e., latent class separation). Both parameters range between 0 and 1; the closer to 1, the better the fit. A value of at least 0.7 is recommended as an indicator of sufficient latent class separation [37], meaning that estimated patterns are substantially different between classes. Apart from these statistical criteria, the theoretical background and interpretability of class solutions were also considered a decision aid in model selection. To assess conditional independence, we checked standardized bivariate residuals for each model, with values exceeding |3.84| indicating conditional dependence [40].

Following model selection, sociodemographic data, behavioral intentions, and sexual behavior variables were compared between latent classes using chi-square (for categorical variables) and one-way ANOVA (for continuous ones). Cramer’s *V* for categorical variables and the partial eta square for continuous variables were calculated as effect size coefficients. Multinomial logistic regression was performed to assess the association between latent classes and adolescents’ sex, age, educational level, sexual experience, and condom use, and relative prevalence ratios (RPR) are reported for significant results. These analyses were conducted with SPSS 25 and based on α = 0.05.

## 3. Results

### 3.1. Latent Class Models

**Figure 1:**
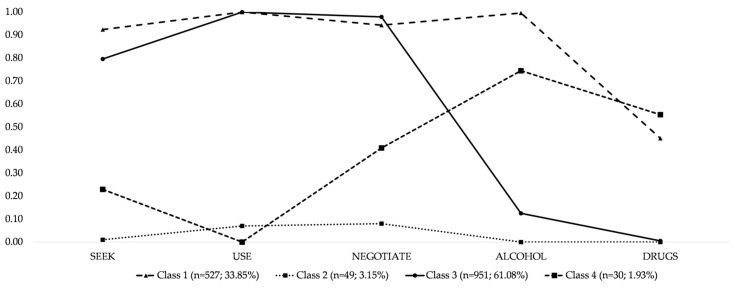
Estimated indicator probabilities for latent classes of behavioral intentions regarding sexual activity (i.e., intentions to seek, negotiate the use of, and use, condoms, as well as combining alcohol or other drug use and sexual activity), ranging from 0 (low probability) to 1 (high probability) in a sample of Spanish adolescents (*N* = 1557).

To identify the best model, statistical criteria and interpretability of classes were considered (see 2.4. Data Analyses for details). Table 2 lists model fit criteria for models with up to five latent classes. A model with six classes was also tested but could not be successfully identified using the selected estimation methods. A model with four latent classes was chosen because most criteria showed the best fit for this model, and entropy (0.88), as well as ALCP (0.81 to 0.98), was also sufficient. An analysis of standardized bivariate residuals revealed values below |0.40|, thus indicating conditional independence. Estimated indicator probabilities for the latent class model are shown in Figure 1.

The first class (“condom + drugs”; *n* = 527) comprises about one-third of the sample and reports moderate to high probabilities for all facets of intention. The second class (“abstinent”; *n* = 49) is small and has distinctively very low probabilities overall. The third class (“condom + no drugs”; *n* = 951) is the largest class (about 61% of the sample) and is characterized by high probabilities for the intention to seek, negotiate, and use condoms, as well as low probabilities of intending to combine alcohol and other drug use and sexual activity. The fourth class (“no condom + drugs”; *n* = 30) is the smallest class and it has low probabilities of intending to seek, negotiate, or use condoms but moderate to high probabilities of mixing alcohol and other drugs with sexual activities. Therefore, class 3 has the lowest risk profile (based on intentions), with classes 1, 2, and 4 mirroring an increase in risk. 

### 3.2. Association between Behavioral Intentions and Latent Classes

Table 3 summarizes the differences in sociodemographic variables and behavioral intention scores by latent classes, based on the results of cross-tables and ANOVA analyses. Latent classes did not differ in socioeconomic status or family situation but presented small to large effects (η^2^ ranged from 0.03 to 0.67) regarding other sociodemographic variables. Adolescents belonging to the “no condom + drugs” group were older (than the “condom + no drugs” group) and scored lower in intention to seek condoms (than the “condom + drugs” group), lower in condom use intention, and lower in intention to negotiate condom use (than the “condom + drugs” and the “condom + no drugs” groups). They also had a higher intention to have sex under the influence of alcohol (than the “abstinent” and the “condom + no drugs” groups), and a higher intention to have sex under the influence of drugs (than the other three groups). Adolescents belonging to the “condom + drugs” group were older (than the “condom + no drugs” group), and the proportion of males was higher (than the “condom + no drugs” group). They reported lower condom use and a lower intention to negotiate condom use (than the “condom + no drugs” group), and a higher intention to have sex under the influence of alcohol and drugs (than the “abstinent” and the “condom + no drugs” groups). Adolescents belonging to the “condom + no drugs” group were slightly younger (than the “condom + drugs”) and only presented a lower intention to seek condoms (than the “condom + drugs” and “no condom + drugs” groups). Adolescents belonging to the “abstinent” group presented a lower intention to seek condoms (than the “condom + drugs” and the “condom + no drugs” groups), a lower intention to use condoms, and a lower intention to negotiate condom use (than the rest).

### 3.3. Predicting Latent Class Membership

Multinomial logistic regression was used to assess the association between class membership and participants’ sociodemographic variables, including sex (female vs. male), age, educational level (categories: “9th grade”, “10th grade”, “curriculum adaptation—level 1”, and “curriculum adaptation—level”), socioeconomic status (categories: “Low”, “medium”, and “high”), family situation (categories: “Married parents”, “divorced parents”, “unmarried parents living together”, “single parent”, and “orphan”), and behavioral variables, including sexual experience (categories: “Yes” vs. “no”), and percentage of condom use (from “0” to “100”).

Results (see Table 4) indicated that, compared to the “condom + drugs” group (Class 1), the “abstinent” group (Class 2) and the “no condom + drugs” group (Class 4) were less likely to use condoms when they had sex; in addition, the “condom + no drugs” group (Class 3) included a higher proportion of women.

Compared to the “abstinent” group (Class 2), the proportion of sexually experienced adolescents was higher in the “condom + drugs” (Class 1) and the “condom + no drugs” groups (Class 4), but it was lower in the “condom + no drugs” group (Class 3). The percentage of condom use was higher in the “condom + drugs” (Class 1) and the “condom + no drugs” groups (Class 3), compared to the “abstinent” group (Class 2).

Compared to the “condom + no drugs” group (Class 3), the proportion of females was lower in the “condom + drugs” (Class 1), and the mean age was higher in the “abstinent” group (Class 2). The percentage of condom use was lower in the “abstinent” (Class 2) and the “no condom + drugs” groups (Class 4), compared to the “condom + no drugs” group (Class 3).

Compared to the “no condom + drugs” group (Class 4), the percentage of condom use was higher in the “condom + drugs” (Class 1) and the “condom + no drugs” groups (Class 3). No differences were observed in the rest of the variables.

## 4. Discussion

In the current study, we examined different patterns of sexual risk in a sample of 1557 Spanish adolescents aged 14–16. Using LCA to categorize behavioral intentions, including the intention to seek condoms, intention to negotiate condom use with the sexual partner, condom use intention, and intention to have sex under the influence of alcohol and drugs, we found four distinct risk profiles. Factors associated with class membership were also explored, considering participants’ sociodemographic variables (sex, age, educational level, socioeconomic status, and family situation), and behavioral variables (sexual experience and percentage of condom use).

Four latent classes are highlighted: “Condom + drugs” (Class 1), “abstinent” (Class 2), “condom + no drugs” (Class 3), and “no condom + drugs” (Class 4). Although only 30 participants (almost 2% of the sample) were classified as “no condom + drugs” (Class 4), this group represented the highest sexual risk. Adolescents in this group had low probabilities of intending to seek condoms, negotiating condom use with the sexual partner, and using condoms, but moderate to high probabilities of mixing alcohol and other drugs with sexual activities. Participants assigned to the “condom + drugs” group (Class 1) represented about one-third of the participants and presented moderate to high probabilities for all dimensions of behavioral intentions related to sexual risk. Adolescents classified as “abstinent” (Class 2; about 3% of the sample) presented distinctively very low probabilities overall. Lastly, participants classified as “condom + no drugs” (Class 3) represented the largest group (about 61% of the sample). According to empirical and theoretical studies [15,21,41], this is possibly the group with the lowest risk of contracting STIs and conceiving unplanned pregnancies because they were more likely to use a condom and less likely to have sex under the influence of alcohol and drugs. Adolescents in this group presented high probabilities for the intention to seek, negotiate, and use condoms, as well as low probabilities of intending to combine alcohol and other drugs with sexual activity.

According to socio-cognitive models of health behavior, adolescents with a lower intention of seeking condoms, negotiating condom use with the sexual partner, and using condoms are less likely to perform these behaviors [15,17,21]. Moreover, having sex under the influence of alcohol and other drugs may increase the probability of engaging in sexual risk behaviors, such as having sex without a condom. For example, Espada et al. [24] found that Spanish adolescents who reported having had sex under the influence of alcohol were less likely to report consistent condom use, despite being more likely to engage in vaginal sex, oral sex, and anal sex, compared to those who reported not having mixed sex and alcohol before. Based on these theoretical and empirical studies, “no condom + drugs” (Class 4) presented the highest sexual risk, while “condom + no drugs” (Class 3) presented the lowest sexual risk. Participants assigned to the “abstinent” (Class 2) and “condom + drugs” (Class 1) groups presented a low to moderate sexual risk, respectively.

There were several differences in participants’ sociodemographic variables by latent classes, except for socioeconomic level and family situation. Females were more likely to belong to the “condom + no drugs” (Class 3) compared to the “condom + drugs” group (Class 1) (59% vs. 31.1%, respectively), which suggests that a higher proportion of females were part of the group with the lowest sexual risk. This is consistent with previous studies that highlighted that females are more likely to present more favorable attitudes toward condom use, and consistently higher condom use intention [21,31]. Therefore, it would be expected that females are more likely to use condoms in their sexual relationships, and present lower sexual risk compared to boys. Older adolescents (and therefore belonging to higher educational levels) were more likely to belong to the “condom + drugs” (Class 1) and “no condom + drugs” (Class 4) groups, compared to the “condom + no drugs” (Class 3) group. This result suggests that, as adolescents get older, they are more likely to engage in sexual behaviors and consume alcohol and drugs. This may explain why older adolescents are more present in groups with a greater intention of combining sex and alcohol, compared to the youngest ones.

Sexual experience and condom use percentage significantly differed by latent classes. Sexually experienced adolescents were more likely to be classified as “condom + drugs” (Class 1) and “no condom + drugs” (Class 4) groups, compared to the “condom + no drugs” group (Class 3). This suggests that initiation in the first sexual experiences may coincide with the consumption of alcohol and other drugs [23,24]. Classes 1 and 4 had similar moderate to high probabilities of having sex under the influence of alcohol and/or other drugs. However, those belonging to Class 1 were more likely to use a condom to protect themselves during sexual intercourse, and those belonging to Class 4 presented a lower percentage of condom use.

Adolescents who reported a higher percentage of condom use in their sexual relationships tended to belong to the “condom + drugs” (Class 1: 86.50%) and “condom + no drugs” (Class 3: 85.60%) groups, compared to the “abstinent” (Class 2: 50%) and “no condom + drugs” (Class 4: 56.42%) groups. In the “abstinent” group, although they reported a low percentage of condom use in their sexual relationships, only 28% of them reported being sexually experienced. As most of them were not sexually experienced, they would not be expected to present a high intention of seeking condoms, negotiating their use, and using them, or to report a high percentage of condom use. Actually, the “abstinent” group is considered the group with the lowest sexual risk, just behind the “condom + no drugs” group (Class 3). Considering that adolescents in the “no condom + drugs” group (Class 4) were less likely to report the intention to seek condoms, negotiate condom use, and use condoms but a higher intention of having sex under the influence of alcohol and drugs, they were considered to present the highest risk of engaging in a risky sexual relationship, such as having unprotected sex. 

This study has some limitations. This sample was not representative of adolescents in Spain. Although the sample is geographically dispersed, there is no representation of all the autonomous communities in Spain. Data were obtained through quantitative self-reports. Although they are the most common form of evaluation in our field of study, they are not exempt from methodological issues, such as social desirability. Future research could address these limitations by using a larger and more representative sample and using qualitative methods to understand adolescents’ motivation to engage in sexual risk practices, such as unprotected sex. Due to the low percentage of sexually experienced participants (only about one-third of the sample), condom use was not included as a classifier variable for latent classes. This allows us to capture a more realistic representation of the behavioral intentions and sexual behavior of Spanish adolescents. If we had selected only sexually active youth, this would have biased our results because, by default, they are at a higher risk than youth who are not active. It would be interesting to replicate this study with a large sample of young people who report being sexually active to study different patterns of sexual risk, considering, in addition to behavioral intentions, the use of condoms as the main method of protection against STIs and unplanned pregnancies in this group. The lack of studies similar to this one made it difficult to compare and discuss the results.

## 5. Conclusions

In conclusion, our findings suggest that the level of sexual risk may vary depending on behavioral intentions associated with protective behaviors, such as condom use, as well as the intention to engage in risky practices, such as having sex under the influence of alcohol and other drugs. In our study, more than half of the adolescents (61%) were classified in Class 3 (“condom + no drugs”) and presented a low level of sexual risk, considering the results of previous empirical studies [23,25] and theoretical models to predict health behaviors [15,21,41]. These participants presented high probabilities of engaging in safe sexual behaviors such as negotiating condom use with their sexual partner and using condoms if they have sexual intercourse, besides a low probability of engaging in sexual risk behaviors such as having sex under the influence of alcohol and other drugs.

Only about one-third of the participants were sexually experienced and reported steady condom use for about 86% of the times they had sex (Class 1: “Condom + drugs”). This is a similar percentage as reported in a previous study with Spanish adolescents aged 15–18 [31]. It was considered that this group—the second largest (33.8%)—presented a moderate level of sexual risk, based on socio-cognitive models widely used to predict healthy behaviors such as condom use [17,21]. On the one hand, these adolescents reported higher probabilities of engaging in safe behaviors—such as having the intention to seek condoms, negotiate condom use with the sexual partner, and use condoms if they have sex—than the other classes. On the other hand, they also reported a higher intention to engage in risky behaviors, such as combining sex and alcohol to a moderate-high extent—which has been associated with other sexual risks such as early sexual initiation and number of sexual partners [25,28]—compared to Classes 2 and 3. Almost half of these individuals were sexually experienced (44.6%), and the percentage of condom use was about 86% of the times they had sex. 

Of the entire sample, only 3.1% of the participants reported a low intention to engage in safe behaviors such as seeking condoms, negotiating condom use, and using a condom, and also a low probability of having sex under the influence of alcohol and drugs. Considering that about 70% of them reported not being sexually experienced, they were classified in the group called “abstinent” (Class 2: “Abstinent”). Due to the low probability of sexually experienced individuals in this group, it is not surprising that the percentage of condom use was low. It would be interesting to explore whether the low intention to engage in the target practices is due to a lack of interest in sexual relationships. 

Finally, almost 2% of the participants were characterized by having moderate-high probabilities of engaging in sexual risk, such as having the intention to have sex while under the influence of alcohol and drugs, and low intention to seek condoms, negotiate its use with the sexual partner, and condom use (Class 4: “No condom + drugs”). The majority of this group were sexually active (60%), and they reported using a condom only half the times they had sex. This makes this group the one with the highest risk of contracting an STI and/or conceiving an unplanned pregnancy, based on the main risk factors included in theoretical models of health behaviors [17,21].

Our study allows us to identify four sexual risk profiles in adolescents, based on their intention to engage in healthy or risky sexual behaviors. Unlike previous studies that analyzed sexual risk based on risk factors [31,32], this work provides a multidimensional perspective from which to delve into the profiles of sexual risk in adolescence. LCA allowed us to examine multiple attitudes simultaneously, which provides a more nuanced perspective on often-conflicting cognitions in adolescents (e.g., personal norms vs. perceived peer pressure regarding drug use or sexual activity). In contrast to singular indices of risk, this type of analysis reveals attitudinal or behavioral patterns that represent more complex constellations to be addressed via targeted health promotion. The results of this study suggest that most adolescents belong to the group that is characterized by presenting a low sexual risk, based on their behavioral intentions (Class 3: “Condom + no drugs”). However, a significant proportion of participants presented a moderate level of sexual risk due to their intention of engaging in sex under the influence of alcohol and drugs, but also of protecting themselves during sexual intercourse by using condoms (Class 1: “Condom + drugs”). Thus, this group might not benefit from health promotion regarding condom use, as their intention is already high, but rather promotion regarding alcohol/drug use surrounding sexual activities, such as parties or dating. Thus, latent class models can be implemented to identify such patterns and lead to targeted recommendations or interventions.

Class 2 (“abstinent”) and Class 4 (“no condom + drugs”) were represented by a small group of participants. This may be because both profiles represent the two extremes of a continuum, ranging from the lowest (Class 2) to the highest (Class 4) level of sexual risk. On the one hand, Class 2 may represent the profile of adolescents who are beginning their intimate relationships, and most of them have not had sexual intercourse; therefore, they inform of a low intention to engage in sexual behaviors (such as condom use or having sex under the influence of alcohol). On the other hand, Class 4 may represent the group of adolescents who are mostly sexually experienced and have the intention to engage in sexual risk behaviors (e.g., combining sex with alcohol or other drugs), but low intention to protect themselves by using condoms.

Sexual risk reduction interventions are often applied in the school setting [42] and in a group format, without considering the different sexual experiences of adolescents or their sexual risk profiles [43]. Highlighting the sexual risk factors associated with each profile of sexual risk among adolescents provides an opportunity to develop tailored and targeted sexual health promotion interventions, messages, and HIV awareness campaigns focusing on individuals at highest risk for an STI and/or unplanned pregnancy. These findings could be useful for the design of a screening tool for detecting sexual risk profiles based on behavioral intentions, even creating an algorithm to predict risky sexual behaviors in this population, as proposed by Dangerfield et al. [44] in a study aimed at analyzing black men who have sex with men, and women’s sexual risk profiles using LCA. Future research in sexual health promotion could benefit from this approach, for instance, with the development of risk profile scales. With a better understanding of adolescents’ behavioral intentions, sexual risk reduction interventions will be more suitable to address the prevention of adolescents’ unplanned pregnancies and STIs.

## Figures and Tables

**Table 1:** Demographic distribution of the sample of Spanish adolescents.

Females, *N* (%)	760 (48.8)
Age, *M* ± *SD*	14.87 ± 1.02
Educational level, *N* (%)	
9th grade	835 (53.6)
10th grade	562 (36.1)
Curriculum adaptation—level 1	88 (5.7)
Curriculum adaptation—level 2	72 (4.6)
Socioeconomic status, *N* (%)	
Low	521 (33.35)
Medium	919 (59)
High	117 (7.5)
Family situation, *N* (%)	
Married parents	1144 (77.1)
Divorced parents	309 (20.8)
Unmarried parents living together	8 (0.5)
Single parent	21 (1.4)
Orphan	1 (0.1)
Behavioral intentions, *M* ± *SD (range: 1–5)*	
seek condoms	3.62 ± 1.36
condom use	4.45 ± 0.96
negotiate condom use	4.27 ± 1.07
have sex under alcohol influence	2.18 ± 1.15
have sex under drugs influence	1.47 ± 0.94
Sexually experienced *, *N* (%)	506 (32.5)
Percentage of condom use, *M* ± *SD*	85.13 (22.65)

*Note*. *M* = Mean; *SD* = Standard Deviation; * reported having had vaginal sex, and/or anal sex and/or oral sex.

**Table 2:** Model fit criteria for latent class models of behavioral intentions regarding sexual (risk) behavior in a sample of Spanish adolescents (*N* = 1557).

	1 Class	2 Classes	3 Classes	4 Classes	5 Classes	6 Classes
Free parameters	5	11	17	23	29	Not identified
BLRT ^1^	-	479.59 ***	423.45 ***	38.96 ***	5.98	
AIC ^2^	6355.82	5888.23	5476.78	**5449.82**	5455.84	
SSABIC ^3^	6366.69	5912.14	5513.74	**5499.81**	5518.88	
Entropy	-	1.00	0.86	0.88	0.89	
ALCP ^4^	1.00	1.00	0.81	0.98	0.81	
		1.00	0.99	0.94	0.90	
			0.98	0.98	0.64	
				0.81	0.98	
					0.90	

^1^ BLRT, bootstrapped likelihood ratio test. ^2^ AIC, Akaike Information Criterion. ^3^ SSABIC, sample-size-adjusted Bayes Information Criterion. ^4^ ALCP, average latent class probabilities. Fit criteria indicating the best model are printed in bold. *** *p* < 0.001.

**Table 3:** Sociodemographic variables and behavioral intentions depending on latent class membership.

	Total	Class 1 “Condom + Drugs” (*n* = 527)	Class 2 “Abstinent” (*n* = 49)	Class 3 “Condom + No Drugs” (*n* = 951)	Class 4 “No Condom + Drugs” (*n* = 30)	*F*/χ^2^	E.S.	Direction
Sociodemographics								
Females, *N* (%)	760 (48.8)	164 (31.1)	25 (51)	561 (59)	10 (33.3)	108.42 ***	0.26	3 > 1
Age, *M* ± *SD*	14.87 ± 1.02	15.12 ± 1.04	14.82 ± 0.95	14.71 ± 0.98	15.30 ± 1.23	20.22 ***	0.03	1 > 3 4 > 3
Educational level, *N* (%)								
9th grade	835 (53.6)	243 (46.1)	30 (61.2)	550 (57.8)	12 (40)	58.37 ***	0.11	3 > 1
10th grade	562 (36.1)	204 (38.7)	14 (28.6)	335 (35.2)	9 (30)			-
Curriculum adaptation—level 1	88 (5.7)	34 (6.5)	4 (8.2)	45 (4.7)	5 (16.7)			4 > 3
Curriculum adaptation—level 2	72 (4.6)	46 (8.7)	1 (2)	21 (2.2)	4 (13.3)			4 > 3 4 > 1
Socioeconomic status, *N* (%)								
Low	521 (33.35)	172 (32.6)	19 (38.8)	317 (33.3)	13 (43.3)	4.84	-	-
Medium	919 (59)	307 (58.3)	27 (55.1)	570 (59.9)	15 (50)			
High	117 (7.5)	48 (9.1)	3 (6.1)	64 (6.7)	2 (6.7)			
Family situation, *N* (%)								
Married parents	1144 (77.1)	382 (76.2)	36 (73.5)	709 (78.3)	17 (60.7)	15.95	-	-
Divorced parents	309 (20.8)	109 (21.8)	13 (26.5)	176 (19.4)	11 (39.3)			
Unmarried parents living together	8 (0.5)	0 (0)	0 (0)	8 (0.9)	0 (0)			
Single parent	21 (1.4)	10 (2)	0 (0)	11 (1.2)	0 (0)			
Orphan	1 (0.1)	0 (0)	0 (0)	1 (0.1)	0 (0)			
Behavioral intentions, *M* ± *SD*								
Seek condoms	3.62 ± 1.36	4.10 ± 0.95	1.20 ± 0.40	1.83 ± 0.98	3.62 ± 1.36	111.10 ***	0.17	1 > 2 1 > 3 1 > 4 3 > 2 4 > 3
Condom use	4.45 ± 0.96	4.46 ± 0.75	1.20 ± 0.40	4.68 ± 0.60	1.76 ± 0.43	610.22 ***	0.54	1 > 2 3 > 1 1 > 4 3 > 2 4 > 2 3 > 4
Negotiate condom use	4.27 ± 1.07	4.19 ± 0.97	1.37 ± 0.72	4.53 ± 0.80	2.13 ± 1.07	269.08 ***	0.34	1 > 2 3 > 1 1 > 4 3 > 2 4 > 2 3 > 4
Have sex under alcohol influence	2.18 ± 1.15	3.45 ± 0.70	1.16 ± 0.37	1.50 ± 0.62	3.30 ± 1.14	1069.65 ***	0.67	1 > 2 1 > 3 3 > 2 4 > 2 4 > 3
Have sex under drugs influence	1.47 ± 0.94	2.06 ± 1.24	1.14 ± 0.35	1.12 ± 0.36	2.87 ± 1.54	184.23 ***	0.26	1 > 2 1 > 3 4 > 1 4 > 2 4 > 3
Sexually experienced *, *N* (%)	506 (32.5)	235 (44.6)	14 (28.6)	239 (25.1)	18 (60)	69.35 ***	0.21	4 > 1 4 > 3 1 > 3
Percentage of condom use, *M* ± *SD*	85.13 (22.65)	86.50 (20.05)	50 (33.41)	85.60 (23.18)	56.42 (36.82)	7.59 ***	0.05	1 > 2 1 > 4 3 > 2 3 > 4

*Note*. *M* = Mean; *SD* = Standard Deviation; *F* = Fisher’s *F-*test for quantitative variables; χ^2^ = chi-square for sex (categorical variable); *p* = *p*-value; E.S. = eta squared for continuous variables and Cramer’s *V* for categorical variables. * reported having had vaginal, anal, and/or oral sex sexual practices; *** *p* < 0.001.

**Table 4:** Results of multinomial logistic regression predicting latent class membership by sociodemographic and behavioral variables.

	Class 1 “Condom + Drugs”	Class 2 “Abstinent”	Class 3 “Condom + No Drugs”	Class 4 “No Condom + Drugs”
	*n* = 527	*n* = 49	*n* = 951	*n* = 30
	RPR [95% CI]	RPR [95% CI]	RPR [95% CI]	RPR [95% CI]
**Reference: Class 1**				
Sex	-	2.36 [0.16, 33.95]	0.29 [0.18, 0.47] ***	1.69 [0.17, 16.28]
Age	-	0.48 [0.12, 1.83]	0.89 [0.70, 1.15]	0.49 [0.19, 1.28]
Educational level	-			
1 = 9th grade	-	1.30 [0, 2.30]	0.93 [0.47, 1.86]	0.27 [0.02, 2.74]
2 = 10th grade	-	1.92 [0.16, 22.09]	0.97 [0.54, 1.74]	0.22 [0.02, 2.50]
3 = ref.	-	-	-	-
Sexual experience	-	3.23 [0]	0.79 [0.31, 2.02]	1.22 [0.11, 12.75]
Condom use	-	0.95 [0.91, 0.98] **	0.99 [0.98, 1.01]	0.96 [0.93, 0.98] **
**Reference: Class 2**				
Sex	0.42 [0.02, 6.09]	-	0.12 [0.009, 1.81]	0.72 [0.02, 21.96]
Age	2.05 [0.54, 7.75]	-	1.85 [0.49, 6.97]	1.01 [0.20, 5.05]
Educational level		-		
1 = 9th grade	>99 ***^a^	-	>99 ***^a^	>99 ***^a^
2 = 10th grade	0.51 [0.04, 5.95]	-	0.50 [0.04, 5.86]	0.11 [0.004, 3.26]
3 = ref.	-	-	-	-
Sexual experience	>99 ***^a^	-	>99 ***^a^	>99 ***^a^
Condom use	1.05 [1.01, 1.09] **	-	1.05 [1.01, 1.09] **	1.01 [0.20, 5.05]
**Reference: Class 3**				
Sex	3.34 [2.11, 5.29] ***	7.90 [0.55, 113.18]	-	5.68 [0.59, 54.17]
Age	1.11 [0.86, 1.42]	0.95 [0.91, 0.98] **	-	0.55 [0.21, 1.43]
Educational level			-	
1 = 9th grade	1.06 [0.53, 2.11]	1.39 [0]	-	0.29 [0.03, 2.96]
2 = 10th grade	1.02 [0.57, 1.84]	1.97 [0.17, 22.95]	-	0.23 [0.02, 2.59]
3 = ref.	-	-	-	-
Sexual experience	1.26 [0.49, 3.22]	4.08 [4.08, 4.08]	-	1.55 [0.14, 16.68]
Condom use	1 [0.99, 1.01]	0.95 [0.91, 0.98] **	-	0.96 [0.94, 0.98] **
**Reference: Class 4**				
Sex	0.58 [0.06, 5.64]	1.39 [0.04, 42.43]	0.17 [0.01, 1.67]	-
Age	2.02 [0.77, 5.27]	0.98 [0.19, 4.89]	1.03 [1.01, 1.06]**	-
Educational level				-
1 = 9th grade	3.58 [0.36, 35.22]	4.69 [0]	3.36 [0.33, 33.61]	-
2 = 10th grade	4.36 [0.40, 47.68]	8.41 [0.30, 230.76]	4.25 [0.38, 46.89]	-
3 = ref.	-	-	-	-
Sexual experience	0.81 [0.07, 8.47]	2.63 [2.63, 2.63]	0.64 [0.06, 6.94]	-
Condom use	1.03 [1.01, 1.06] **	0.98 [0.19, 4.89]	1.03 [1.01, 1.06] **	-

*Note.* ref = reference class; RPR = relative prevalence ratio; CI = confidence interval; significant results are printed in bold. ** *p* < 0.01, *** *p* < 0.001. ^a^ The prevalence of the reference category was very low in the reference class, leading to very high RPR estimates, which we have listed as >99.

## Data Availability

The data presented in this study are available on request to the authors.
